# Burosumab treatment in an adult with FGF23-mediated hypophosphatemia due to cutaneous skeletal hypophosphatemia syndrome

**DOI:** 10.1210/jendso/bvag040

**Published:** 2026-03-27

**Authors:** Laura L Tosi, Elmer N Rajah, Austin P Gillies, Mirini Kim, Kendall Reid, Rachel I Gafni

**Affiliations:** Bone Health Program, Division of Orthopaedics & Sports Medicine, Children's National Hospital, Washington, DC 20010, USA; Bone Health Program, Division of Orthopaedics & Sports Medicine, Children's National Hospital, Washington, DC 20010, USA; Bone Health Program, Division of Orthopaedics & Sports Medicine, Children's National Hospital, Washington, DC 20010, USA; Bone Health Program, Division of Orthopaedics & Sports Medicine, Children's National Hospital, Washington, DC 20010, USA; Bone Health Program, Division of Orthopaedics & Sports Medicine, Children's National Hospital, Washington, DC 20010, USA; Bone Health Program, Division of Orthopaedics & Sports Medicine, Children's National Hospital, Washington, DC 20010, USA; National Institute of Dental and Craniofacial Research, National Institutes of Health, Bethesda, MD 20892, USA

**Keywords:** osteomalacia, rickets, hypophosphatemia, fibroblast growth factor 23

## Abstract

**Context:**

Cutaneous skeletal hypophosphatemia syndrome (CSHS) is an ultrarare disorder defined by epidermal and/or melanocytic nevi, mosaic skeletal dysplasia, and FGF23-mediated hypophosphatemia. As in other FGF23-mediated hypophosphatemia conditions, individuals with CSHS have renal phosphate wasting and inappropriately normal or frankly low 1,25-dihydroxyvitamin D levels with resultant hypophosphatemia leading to rickets and osteomalacia. Conventional therapy for FGF23-mediated hypophosphatemia consists of multiple daily doses of oral phosphate and active vitamin D.

**Objective:**

Burosumab is a fully human immunoglobulin G1 monoclonal antibody that binds to and inhibits the activity of FGF23, leading to an increase in serum phosphorus levels and skeletal healing. Given its efficacy in tumor-induced osteomalacia and X-linked hypophosphatemic rickets, two related disorders of FGF23-mediated hypophosphatemia, we explored treatment with burosumab in a young adult with CSHS.

**Methods:**

In this open-label, single-patient trial conducted in the clinical research unit of an academic children's hospital, burosumab was administered subcutaneously every 4 weeks for 3 years. The participant was an 18-year-old woman with CSHS and FGF23-mediated hypophosphatemia. Burosumab was administered subcutaneously every 4 weeks, starting at 0.3 mg/kg/dose and increasing up to 0.9 mg/kg/dose. Main outcome measures included change in blood phosphorus levels.

**Results:**

Burosumab therapy was well tolerated with correction of hypophosphatemia and improvement in other measures including renal phosphate loss, alkaline phosphatase, active vitamin D metabolism, skeletal imaging, pain, physical function, and overall quality of life. Adverse events were manageable, with unclear relationship to burosumab treatment.

**Conclusion:**

These findings suggest that burosumab may be an effective treatment for CSHS.

Cutaneous skeletal hypophosphatemia syndrome (CSHS) is an ultrarare mosaic disorder caused by postzygotic somatic variants in *HRAS* and *NRAS* [1]. Affected individuals typically experience epidermal and/or melanocytic nevi and an associated mosaic skeletal dysplasia, as well as an increased risk of other tumors. In a murine model of the disease, it has been shown that the dysplastic bone produces excess fibroblast growth factor 23 (FGF23) [2], a key regulator of phosphate and vitamin D metabolism. As such, individuals with CSHS may experience FGF23-mediated hypophosphatemia, similar to patients with fibrous dysplasia/McCune-Albright syndrome (FD/MAS), another mosaic disease caused by somatic activating variants in GNAS leading to overproduction of FGF23 by dysplastic bone. As in seen (FD/MAS), the hypophosphatemia may remit in some, but not all, cases of CSHS during adulthood [3-5]. While the cause for the selective remission is unknown, it has been suggested to be due to age-related apoptosis and/or senescence of *RAS*-variant bearing bone cells [3, 5]. Other well-known causes of FGF23-mediated hypophosphatemia include tumor-induced osteomalacia (TIO) and X-linked hypophosphatemia (XLH). In all 3 conditions, high circulating levels of FGF23 suppress renal phosphate reabsorption and 25-hydroxyvitamin D-1 α-hydroxylase activity while increasing 24-hydroxylase activity, leading to hypophosphatemia, and decreased 1,25-dihydroxyvitamin D (1,25[OH]2D) production. Across these disorders, the clinical symptoms are similar and often include diffuse skeletal osteomalacia (and, in children, rickets), muscle weakness, fatigue, bone pain, and fractures [6].

Conventional therapy of FGF23-mediated hypophosphatemia includes multiple daily doses of oral phosphorus and active vitamin D in an attempt to maintain normal mineral homeostasis. However, in many patients this treatment is inadequate and is associated with complications such as gastrointestinal distress, hypercalcemia, nephrocalcinosis, and secondary/tertiary hyperparathyroidism [7]. Burosumab is a fully human immunoglobulin G1 monoclonal antibody that binds to and inhibits the activity of FGF23, leading to an increase in serum phosphorus levels [8]. At the initiation of this study, burosumab had been US Food and Drug Administration approved for treatment of XLH. In single- and repeat-dose clinical studies in individuals with XLH, subcutaneous (SC) administration of burosumab consistently increased and sustained serum phosphorus levels and tubular reabsorption of phosphate (TRP) and improved radiologic rickets, without a major effect on urine calcium levels [9-13]. In 2020, burosumab was approved for the treatment of patients with TIO [7], in whom definitive cure by surgical resection or tumor ablation was not possible.

We hypothesized that burosumab would provide clinical benefit to a young adult with persistent FGF23-mediated hypophosphatemia due to CSHS, given the common underlying pathophysiology observed in XLH and TIO.

## Materials and methods

### Patient description

**Figure 1:**
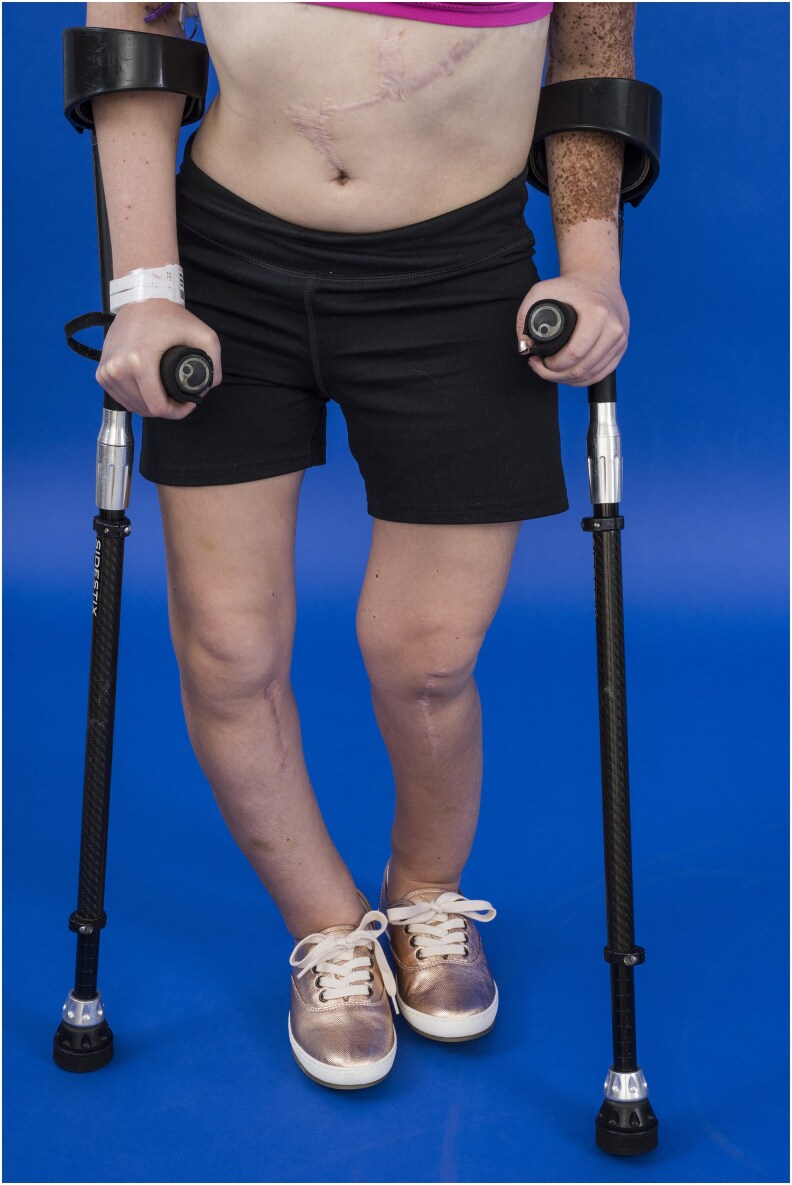
Photograph of the patient at age 17 years. Lower-extremity malalignment and resultant functional limitation from a combination of diffuse hypophosphatemic rickets and left-sided skeletal dysplasia. Epidermal nevi are limited to the left side of the body.

At study enrollment, the patient was an 18-year-old woman with CSHS, manifested by FGF23-mediated hypophosphatemic rickets, left-sided skeletal dysplasia, and epidermal nevi (Fig. 1). A confirmed pathogenic *HRAS* variant in her affected skin and bone was previously reported; additional exome sequencing in this patient did not identify germline variants in any genes implicated in disorders of phosphate metabolism. Biopsies of her affected skin were negative for FGF23 expression by immunohistochemistry [1].

Her history was significant for hypophosphatemia and elevated alkaline phosphatase (ALP) levels diagnosed early in life that were difficult to manage with phosphate and calcitriol. At age 12 years, she developed kidney stones and an elevated parathyroid hormone (PTH) and high normal blood and urine calcium, without preceding secondary hyperparathyroidism. Calcitriol and phosphate replacement were decreased, resulting in the resurgence of symptoms including hypophosphatemia, bone pain, and difficulty ambulating. Treatment with medications such as cinacalcet and hydrochlorothiazide were attempted but she did not tolerate these medications well. Renal ultrasound demonstrated multiple small nonobstructive kidney stones. She was ultimately diagnosed with primary hyperparathyroidism due to a single parathyroid adenoma, which was resected at age 17 years with normalization of PTH and blood and urine calcium. Despite this, serum phosphorus levels remained below the normal limit with an elevated C-terminal FGF23 of 224 RU/mL (normal <180, Mayo Clinic Laboratories) at the screening visit. While intact FGF23 assays were not offered commercially prior to burosumab initiation, previous intact FGF23 levels measured in a research laboratory were also elevated. Although her energy level was reported to have improved mildly after cure of primary hyperparathyroidism, over time she developed increased reliance on a wheelchair for mobility. Medications at the time of study enrollment included calcitriol 0.75 mcg daily divided into twice-daily doses and phosphate supplementation of 250 mg 4 times daily.

**Figure 2:**
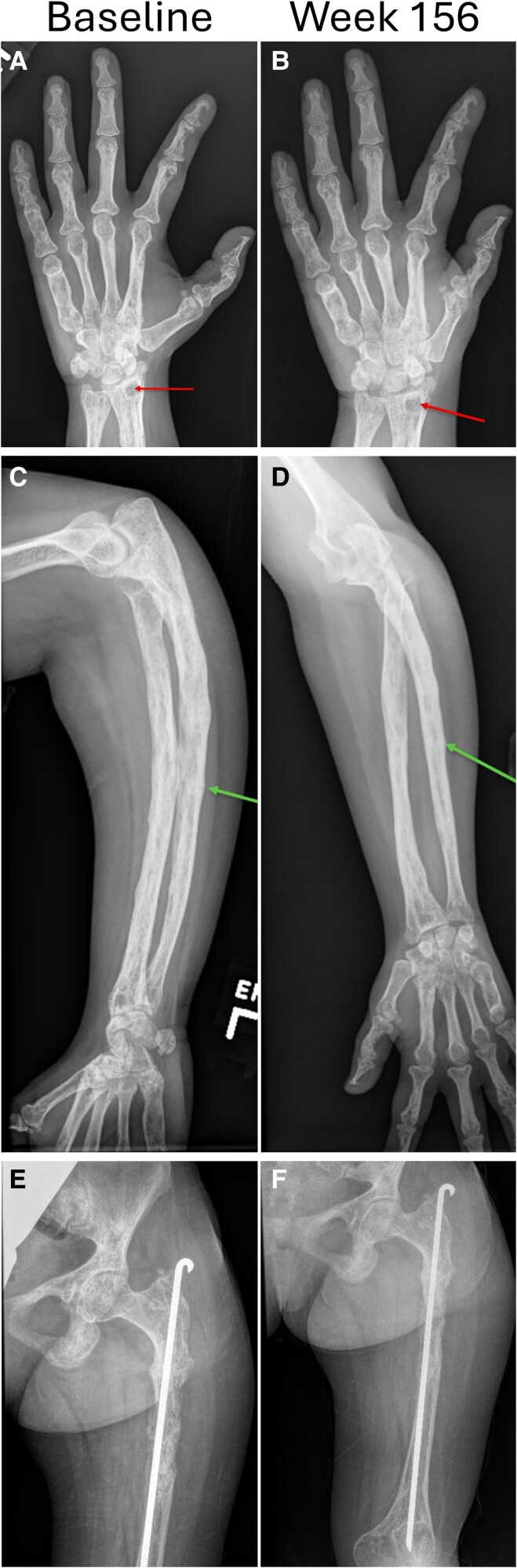
Radiographs at baseline and after 156 weeks of burosumab of the A and B, left hand/wrist; C and D, forearm; and E and F, pelvis/femur. At baseline there was noticeable bony heterogeneity with areas of sclerosis and bone resorption; intramedullary and cortical cysts are present (arrow). With burosumab treatment, bone mineralization improved with reduction in cortical irregularity and resolution of the ulnar fracture (arrow). The cortical cyst remained unchanged.

Due to the combination of the primary skeletal dysplasia and chronic hypophosphatemia, the patient developed bilateral bowed legs at a young age and suffered multiple fractures on her left side, including left tibia, left femur, and left arm. Despite rodding, her left femur and tibia radiographs suggested chronic nonunion or pseudofracture sites. She underwent surgery to correct her bowed tibias with partial success and was monitored for progression of her scoliosis. At study initiation, skeletal radiographs showed closed growth plates with severe disease in her long bones, including metatarsals and metacarpals, with probable nonunion sites, primarily on her left side (Fig. 2). Scoliosis measuring 40° was also present.

Other medical issues include large nevi involving the left side of her face, left arm, left leg, and chest. One of the facial lesions transformed into a squamous cell carcinoma, which was resected without issue at age 15 years. Additionally, she developed seizures at age 1 year and was treated with antiepileptics for about 5 years. Later, as a preteen, she experienced several absence-like events; detailed neurologic work-up at that time was negative. A brain magnetic resonance imaging scan (MRI) performed at age 16 years did not identify any tumors. There was no history of iron deficiency or anemia.

### Trial design

This was a single-site, single-patient, open-label, investigator-initiated trial conducted at the Children's National Research Institute, Washington, DC, USA (NCT03993821). This study was approved by the institutional review board, and the patient provided written informed consent. The patient was concurrently enrolled in an institutional review board–approved natural history study at the National Institutes of Health (NCT00024804).

One week after discontinuation of phosphate and calcitriol supplementation, the participant was administered burosumab subcutaneously every 4 weeks, at a starting dose of 0.3 mg/kg, with subsequent adjustments made to maintain a predose blood phosphorus level within the normal range. The maximum dose allowed in this protocol was 2.0 mg/kg or 90 mg. The initial study design was limited to 52 weeks of treatment; however, given the individual's excellent response detailed later, the study was extended for an additional 2 years.

The primary outcome was a change in blood phosphorus levels. Secondary measures included change in 1,25(OH)2D, ratio of renal TmP reabsorption rate to glomerular filtration rate (TmP/GFR), and ALP. Fasting biochemistries were assessed every 4 weeks, for weeks 0 to 104 and then every 12 weeks in weeks 108 to 156 measuring serum calcium, phosphorus, creatinine, ALP, intact PTH, 1,25(OH)2D, and urine phosphorus, creatinine, and calcium. Additionally, fasting biochemistries were measured at 2 weeks after dosing at weeks 2, 14, and 18, to ensure the absence of hyperphosphatemia. TmP/GFR was calculated using a 2-hour urine phosphate and creatinine collection with 1-hour mid-point blood draw. As this is a single-patient investigational, statistical analyses were not performed.

Additional assessments included functional testing (6-minute walk test, 6MWT; sit-to-stand test), Patient-Reported Outcome Measurement Information System (PROMIS) questionnaires evaluating pain, fatigue, and physical functioning, Brief Pain and Fatigue Inventory, and 36-Item Short Form Health Survey (SF-36) every 6 months.

Exploratory end points included annual bone densitometry by dual-energy x-ray absorptiometry (DXA, Hologic), radiographic skeletal survey, and Technetium Tc 99m diphosphonate bone scan.

## Results

### Burosumab dosing

**Figure 3:**
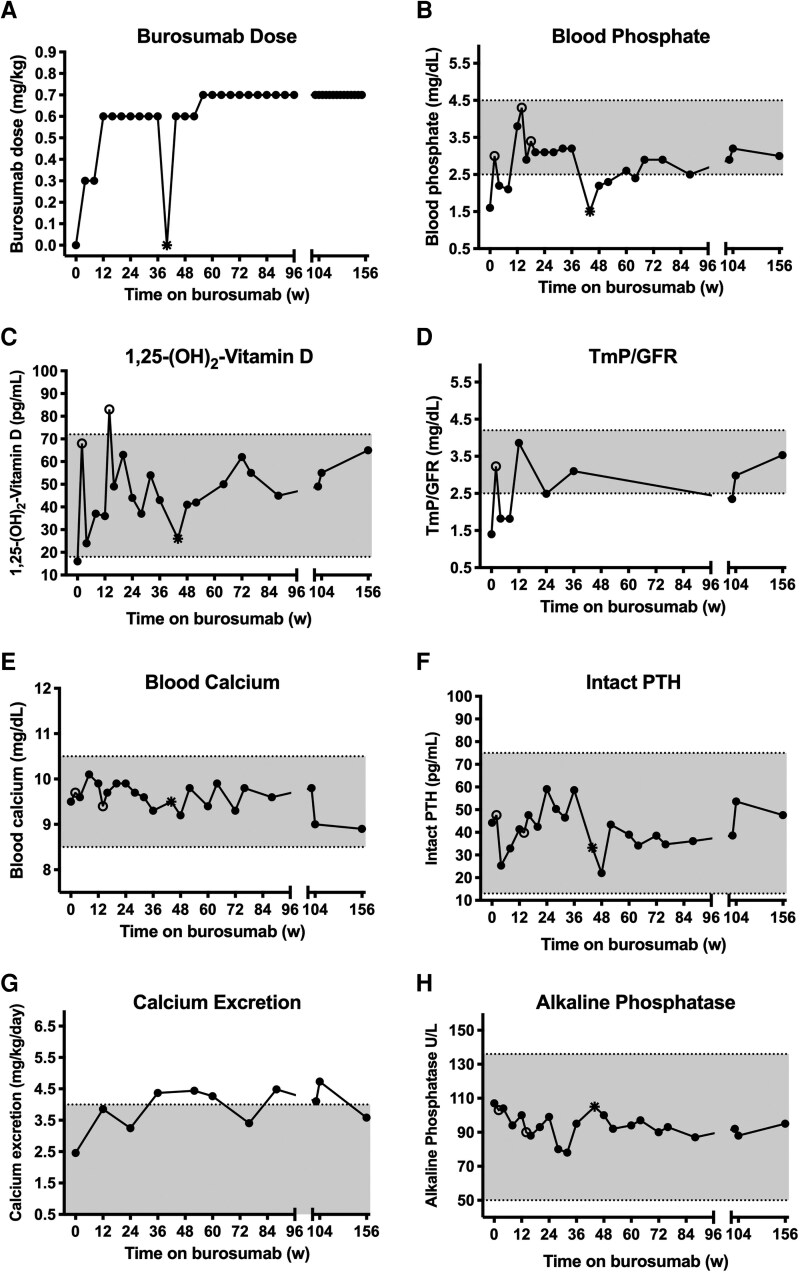
Biochemical response to burosumab therapy given every 4 weeks. A, Burosumab dose; B, blood phosphate; C, 1,25-dihydroxyvitamin D; D, tubular maximum reabsorption of phosphate/glomerular filtration rate (TmP/GFR); E, blood calcium; F, blood intact parathyroid hormone (PTH); G, 24-hour urine calcium excretion for weight; and H, blood alkaline phosphatase before and on 156 weeks of burosumab treatment. Open circles reflect biochemical sampling 2 weeks after dosing at weeks 2, 14, and 18. Closed circles reflect sampling just prior to dosing. TmP/GFR was calculated using a 2-hour urine phosphate and creatinine collection with 1-hour mid-point blood draw. The asterisk reflects a missed dose at week 40 with sampling at week 44—this was 8 weeks after the week 36 dose. All specimens were collected in the fasted state.

Burosumab was administered in the research clinic as an SC injection every 4 weeks for 156 weeks. The starting dose was 0.3 mg/kg. This was increased to 0.6 mg/kg at week 8, 0.7 mg/kg at week 56, and 0.9 mg/kg at week 140 (Fig. 3). The dose was titrated to achieve a fasting, predose serum phosphorus level in the range of 2.5 to 4.0 mg/dL. Alternating sites of administration (abdomen, upper arm, thigh, or buttocks) were used to minimize potential injection site reactions. The required dose level never exceeded 1.5 mL in volume, thus a single injection per administration was sufficient.

Dosing and assessments were missed at week 40 due to the COVID-19 pandemic; all other therapy and evaluations were delivered as planned. The serum phosphorus level did not exceed the upper limit of normal at any time point, thus burosumab was never withheld or down-titrated for this reason.

### Biochemical response

Baseline laboratory values prior to the first dose of burosumab were consistent with FGF23-mediated hypophosphatemia (Table 1 and Fig. 3). Two weeks after the first dose of burosumab, blood phosphorus, 1,25(OH)2D, and TmP/GFR had increased into the normal range. With increments in dosing to maintain fasting, predose blood phosphorus within the normal range, as described earlier, burosumab treatment successfully maintained these parameters mostly within the normal range over 156 weeks. In particular, the response in 1,25(OH)2D was quite robust when measured 2 weeks following the dose at weeks 2 and 14, consistent with restoration of 25-hydroxyvitamin D-1 α-hydroxylase activity. When the COVID pandemic prohibited the patient from coming to the research clinic for testing and dose administration at week 40, her phosphorus level had dropped to 1.5 mg/dL at the week 44 visit, which was 8 weeks after the last dose. Burosumab was restarted although dose increases were required at subsequent visits to maintain predose phosphorus levels within the normal range for the duration of the study. While 1,25(OH)2D levels also decreased following the missed burosumab dose, reflecting increased FGF23 activity, they never became frankly low. ALP remained within the normal range throughout; however, it did decrease from 107 U/L pre burosumab to a nadir of 78 U/L at 32 weeks, rising back to baseline at week 44 following the missed dose, then decreasing again with burosumab resumption. Urinary calcium excretion was intermittently elevated, despite normal PTH and serum calcium throughout.

**Table 1:** Baseline laboratory values prior to initiation of burosumab

Parameter	Value	Reference range
Serum calcium, mg/dL	9.5	8.5-10.5
Serum phosphorus, mg/dL	1.6	2.5-4.5
Serum creatinine, mg/dL	0.47	0.5-1.09
ALP, unit/L	107	50-136
Intact PTH, pg/mL	44.2	13-75
1,25(OH)2D, pg/mL	16	18-72
C-terminal FGF23, RU/mL	224	<180
TmP/GFR, mg/dL	1.4	2.5-4.2
Urine calcium excretion, mg/kg/d	2.5	<4

Abbreviations: 1,25(OH)2D, 1,25-dihydroxyvitamin D; ALP, alkaline phosphatase; FGF23, fibroblast growth factor-23; TmP/GFR, tubular maximum reabsorption of phosphate/glomerular filtration rate; PTH, parathyroid hormone.

### Imaging

**Figure 4:**
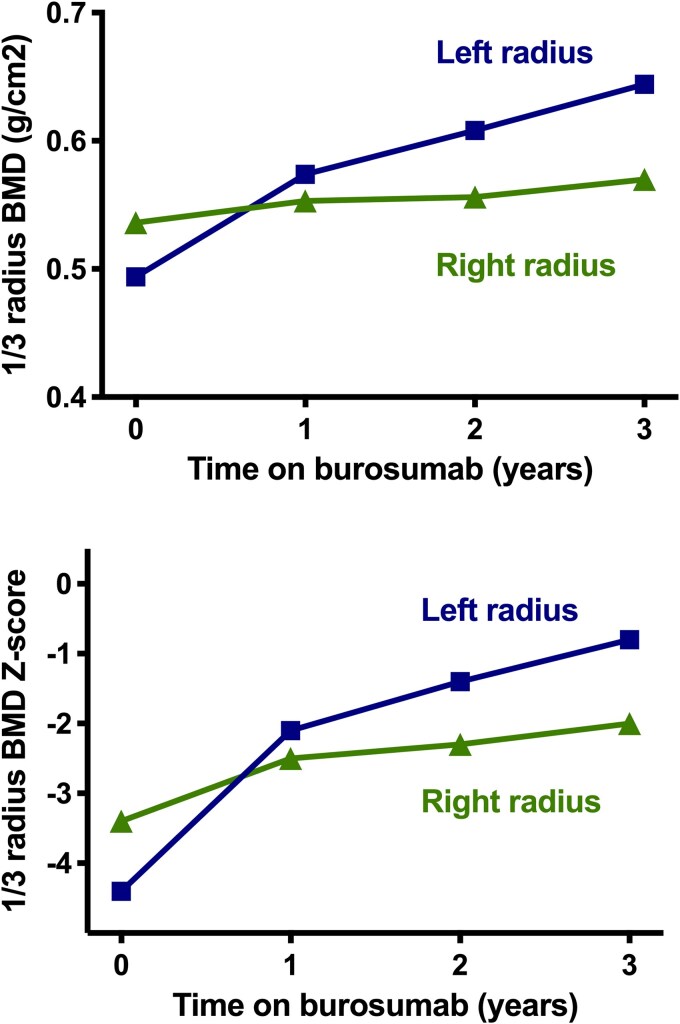
Dual-energy x-ray absorptiometry (DXA) changes in the one-third radius. Areal bone mineral density (top panel) and *Z*-score (bottom panel) increased more notably in the dysplastic left radius than in nondysplastic right radius.

Nonunion/pseudofracture sites were noted to slowly disappear on radiographs over the course of the study (see Fig. 2). DXA scans were limited by the patient's femoral hardware and scoliosis, thus only the distal radius DXA site was interpretable. While only a small amount of change was noted in the bone density of her right (nondysplastic) forearm, a 30.4% increase was noted on her more involved left side (Fig. 4). Her scoliosis remained stable. Technetium bone scans, performed annually, demonstrated stable heterogeneous increased uptake, primarily on the left side of the body. This persistently increased skeletal activity in the dysplastic bone is the likely driver of the asymmetric increase in radial bone accrual seen on DXA. No lesions were noted to disappear, and no new focal abnormalities were seen over the course of the 3-year study.

### Quality of life and physical function

**Figure 5:**
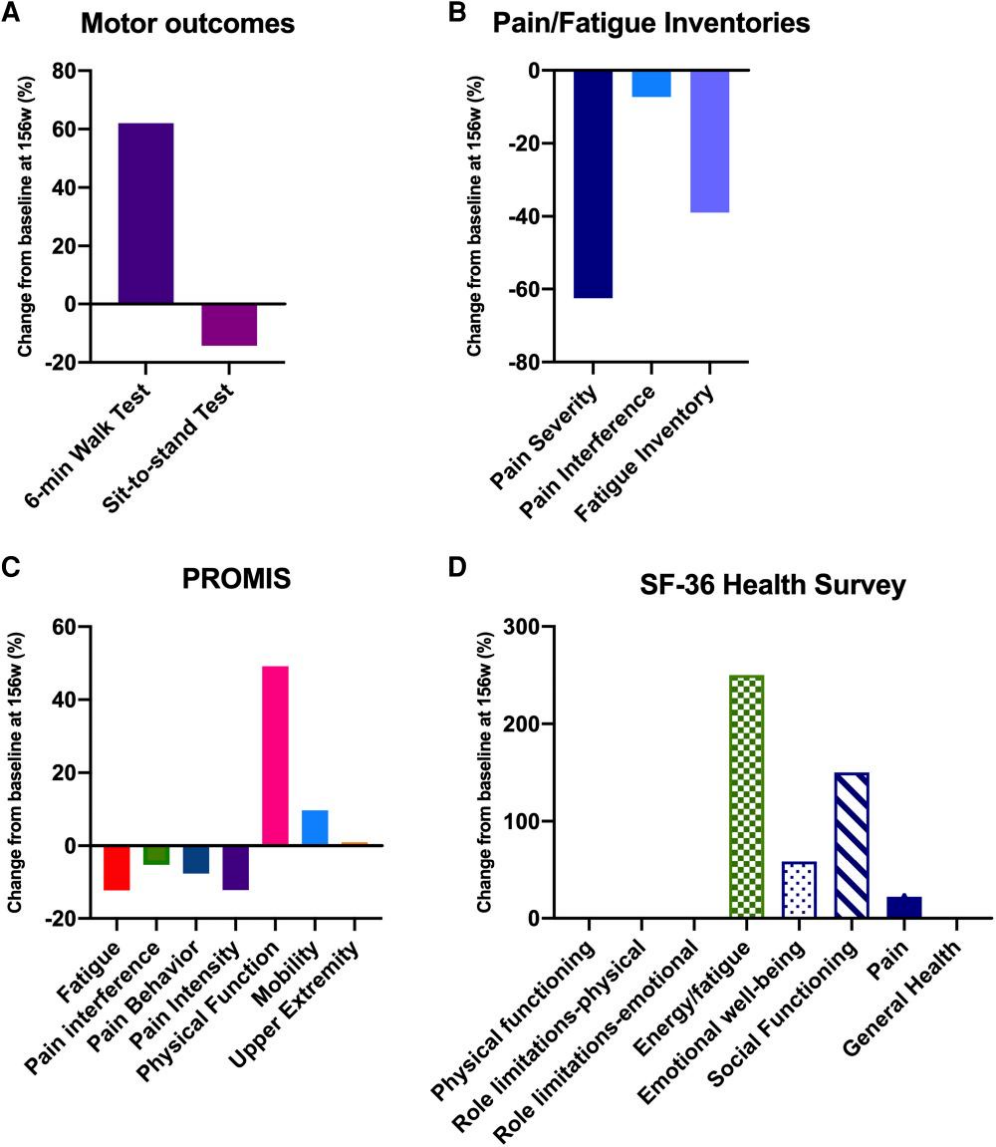
Percentage improvement compared to baseline in patient-reported and motor outcomes after 156 weeks of burosumab. A, Increased distance was achieved in the 6-minute walk test and B, pain and fatigue scales improved. Improved physical function was demonstrated through C, Patient-Reported Outcome Measurement Information System (PROMIS) measures and subjective reports from the patient. D, In the 36-Item Short Form Health Survey (SF-36), improvement was seen in some domains of physical and emotional well-being, while remaining unchanged in some.

At 156 weeks, the patient demonstrated a marked improvement in the 6-minute walk test (Fig. 5A). No change was seen in the sit-to-stand test; however, this may be attributed to researcher error, as the final evaluation was performed after a long day of testing. With the initiation of burosumab, the patient reported reduction in her fatigue and steady improvement in her energy level and physical function (Fig. 5B-5D), such that wheelchair use was limited to when traveling long distances outside the home. On report, her balance has improved enabling her to stand for long periods and participate in outdoor physical activities, such as raking leaves. She particularly appreciated her improved ability to engage socially. As the patient is right-hand dominant, the side unaffected by skeletal dysplasia, upper-extremity function did not appear to change. On the SF-36 (see Fig. 5D), we saw marked improvement in energy/fatigue, emotional well-being, social functioning, as well as minor improvement in pain. General health began within normal limits, and remained there over the course of the study. Role limitations due to emotional problems remained slightly lower than normal throughout the study, and physical functioning and role limitations due to physical health remained significantly lower than normal with no improvement. We used the PROMIS tool Physical Function with a Mobility Aid, which captured the improvement in her independence and mobility, while the SF-36 did not, underscoring the importance of including a functional measure aimed at measuring change in disability status.

### Adverse events

Recurrent symptomatic nephrolithiasis (calcium oxalate) was noted 7 weeks after initiation of burosumab. Given the history of nephrolithiasis and the known presence of nonobstructive stones seen on prestudy ultrasound, the possibility both of increased urine calcium excretion and mobilization of kidney stones by burosumab treatment was considered. Bilateral ureteroscopy and basket stone retrieval with stents were performed in week 28 with resolution of symptoms. Renal ultrasound at the conclusion of the study revealed no evidence of stones.

Approximately 2 years into burosumab therapy, the patient experienced a focal seizure with secondary generalization leading to hospitalization. Electroencephalogram revealed right hemispheric delta theta slowing intermittently and somewhat frequent right frontal temporal sharps in sleep. MRI and computed tomography scans demonstrated mild left mesial temporal sclerosis, asymmetric brain perfusion, a small hyperintense lesion in the right temporal periatrial white matter, and torturous arteries of the circle of Willis. It is unknown if these intracranial findings are due to mosaic *HRAS* variants, as intracranial tumors have been reported in other patients with CSHS [3]. The mesial temporal sclerosis was not seen on the MRI performed at age 16 years; however, that study was less detailed as it was not acquired using an epilepsy protocol. Therefore, it is unknown when the temporal mesial sclerosis developed, as this can be caused by chronic repeated epileptic activity as well as subsequently become a cause of seizures. The patient was started on seizure medications oxcarbazepine and clonazepam as needed. As she had a previous history of seizures and her chemistries were normal at the time of the focal seizure, this was not felt to be related to the burosumab.

Much of her clinical course was punctuated by recurrent severe headaches, mostly in the morning, without clear relationship to timing of the burosumab dose. As hypercalcemia can cause headaches, blood calcium levels were checked several times when she was experiencing headaches; these were always within normal limits. At approximately week 130, she was started on oxcarbazepine, followed by CoQ10 without improvement. However, the initiation of riboflavin (B2) in week 128 led to a profound reduction in headache frequency and severity.

## Discussion

Burosumab treatment in a young adult with FGF23-mediated hypophosphatemia due to CSHS corrected the biochemical effects of excess FGF23, evidenced by normalization of blood phosphate, 1,25(OH)2D, and TmP/GFR. Additionally, she reported a decrease in pain and fatigue with overall improvement in physical and social functioning. Healing of pseudofractures and increase in radial bone density was observed, particularly on the side with dysplastic bone. Burosumab was generally well tolerated, although mobilization of preexisting renal calculi early in treatment may have been related. Urinary calcium excretion was intermittently elevated in the face of normal PTH and serum calcium, the etiology of which remains unclear.

Since this study was initiated, there have been other recent publications demonstrating the effectiveness of burosumab in 4 children with CSHS [14-17]. To our knowledge, there is only one other study treating an adult patient with CSHS, who was included in the TIO study [7] but whose data were published separately [18]. In that report, a 20-year-old man with CSHS was treated with burosumab at a dose of 0.3 mg/kg every 4 weeks for 288 weeks, with normalization of blood phosphorus, 1,25(OH)2D, and TmP/GFR. That patient did not require adjustments in burosumab dose; however, assessments were performed 2 weeks after the dose (ie, “peak levels”), therefore it is unknown how low the phosphorus levels may have dropped in the 2 weeks prior to each dose. This contrasts with our study, in which, with the exception of weeks 2 and 14, we performed assessments immediately pre dose (ie, “trough levels”), with the goal of avoiding hypophosphatemia for the entire 4-week period between doses. We found it necessary to increase the dose of burosumab throughout the course of the study, ultimately reaching 0.9 mg/kg, which is more similar to doses required for patients with XLH and TIO. One possible explanation for the lower dose requirement in the case by Sugarman et al [18] is milder disease, as that patient at baseline exhibited SF-36 measures similar to population norms and less skeletal deformity, fatigue, and pain, with greater and sit-to-stand scores [18] than our patient. In addition, the hypophosphatemia in children with CSHS has been reported to remit spontaneously in some during adulthood [3]. Thus, the improvement in bone scan at week 144 and the bone biopsy without evidence of osteomalacia in that patient at week 48, while on a very low burosumab dose, may also reflect the natural history of the disease. Surprisingly, despite these presumed skeletal improvements, their patient still experienced new vertebral fractures at week 24 that had not healed by week 288; our patient did not experience new fractures over the 156 weeks of treatment. Our patient also showed improvement in PROMIS measures, which were not assessed in the previously published patient.

The primary limitation of this study is that it is a single-patient, uncontrolled study in a highly variable disorder with a wide range of skeletal severity. Furthermore, unlike XLH and TIO, in which the hypophosphatemia is the primary driver of the skeletal disease, individuals with CSHS also have focal areas of skeletal dysplasia, resulting in compounded skeletal fragility at these locations. While improving the overall mineralization by correcting hypophosphatemia and low 1,25(OH)2D appears to be beneficial, even in dysplastic bone, as evidenced by the greater improvement in DXA in our patient's affected arm, further study is needed to fully understand the long-term skeletal benefits of burosumab in adults with CSHS. Additionally, the relationship of burosumab to increasing urinary calcium excretion and potentially mobilizing preexisting kidney stones is unclear. Postmarketing experience with burosumab has suggested that the drug may be associated with alterations in calcium metabolism, including hypercalcemia and increased PTH, leading to a recent change in the manufacturer's labeling.

In this single young adult patient with CSHS and debilitating FGF23-mediated hypophosphatemia, burosumab treatment over 156 weeks corrected phosphorus metabolism and improved skeletal features, pain, fatigue, mobility, and quality of life. While continued study of adults is needed, burosumab appears to be a promising therapeutic option for adult patients with FGF23-mediated hypophosphatemia due to CSHS.

## Data Availability

Original data generated and analyzed during this study are included in this published article or in the data repositories listed in “References.”

## Clinical trial information

ClinicalTrialsID registration number NCT03993821 (registered).

## Abbreviations

1,25(OH)2D: 1,25-dihydroxyvitamin D
ALP: alkaline phosphatase
CSHS: cutaneous skeletal hypophosphatemia syndrome
DXA: dual-energy x-ray absorptiometry
FD/MAS: fibrous dysplasia/McCune-Albright syndrome
FGF23: fibroblast growth factor-23
MRI: magnetic resonance imaging
PROMIS: Patient-Reported Outcome Measurement Information System
PTH: parathyroid hormone
SC: subcutaneous
SF-36: 36-Item Short Form Health Survey
TIO: tumor-induced osteomalacia
TmP/GFR: tubular maximum reabsorption of phosphate/glomerular filtration rate
XLH: X-linked hypophosphatemia
